# The accumulation of Vγ4 T cells with aging is associated with an increased adaptive Vγ4 T cell response after foodborne *Listeria monocytogenes* infection of mice

**DOI:** 10.1186/s12979-022-00275-y

**Published:** 2022-05-03

**Authors:** Camille Khairallah, Timothy H. Chu, Zhijuan Qiu, Jessica N. Imperato, Daniella Yang, Brian S. Sheridan

**Affiliations:** Department of Microbiology and Immunology, Center for Infectious Diseases, Renaissance School of Medicine, Stony Brook University, 246 Centers for Molecular Medicine, Stony Brook, 11794 NY USA

**Keywords:** γδ T cells, Mucosal immunity, Immunosenescence, *Listeria monocytogenes*, Foodborne infection

## Abstract

**Background:**

It is generally accepted that aging has detrimental effects on conventional T cell responses to systemic infections. However, most pathogens naturally invade the body through mucosal barriers. Although mucosal sites are highly enriched in unconventional immune sentinels like γδ T cells, little is currently known about the impact of aging on unconventional mucosal T cell responses. We previously established that foodborne infection with a mouse-adapted internalin A mutant *Listeria monocytogenes* (*Lm*) generates an adaptive intestinal memory CD44^hi^ CD27^neg^ Vγ4 T cells capable of co-producing IL-17A and IFNγ. Therefore, we used this model to evaluate the impact of aging on adaptive Vγ4 T cell responses elicited by foodborne infection.

**Results:**

Foodborne *Lm* infection of female Balb/c and C57BL/6 mice led to an increased adaptive CD44^hi^ CD27^neg^ Vγ4 T cell response associated with aging. Moreover, *Lm*-elicited CD44^hi^ CD27^neg^ Vγ4 T cells maintained diverse functional subsets despite some alterations favoring IL-17A production as mice aged. In contrast to the documented susceptibility of aged mice to intravenous *Lm* infection, mice contained bacteria after foodborne *Lm* infection suggesting that elevated bacterial burden was not a major factor driving the increased adaptive CD44^hi^ CD27^neg^ Vγ4 T cell response associated with mouse age. However, CD44^hi^ CD27^neg^ Vγ4 T cells accumulated in naïve mice as they aged suggesting that an increased precursor frequency contributes to the robust *Lm*-elicited mucosal response observed. Body mass did not appear to have a strong positive association with CD44^hi^ CD27^neg^ Vγ4 T cells within age groups. Although an increased adaptive CD44^hi^ CD27^neg^ Vγ4 T cell response may contribute to foodborne *Lm* resistance of C57BL/6 mice aged 19 or more months, neither anti-TCRδ or anti-IL-17A treatment impacted *Lm* colonization after primary infection. These results suggest that γδTCR signaling and IL-17A are dispensable for protection after primary foodborne *Lm* infection consistent with the role of conventional T cells during the early innate immune response to *Lm*.

**Conclusions:**

*Lm*-elicited adaptive Vγ4 T cells appear resistant to immunosenescence and memory Vγ4 T cells could be utilized to provide protective immune functions during enteric infection of aged hosts. As such, oral immunization might offer an efficient therapeutic approach to generate unconventional memory T cells in the elderly.

**Supplementary information:**

The online version contains supplementary material available at 10.1186/s12979-022-00275-y.

## Background

Immunosenescence is thought to be a major contributor to the heightened susceptibility of the elderly to infection [1]. Moreover, it is also associated with reduced vaccine effectiveness, compounding the seriousness of this issue and limiting therapeutic options for the elderly. Generally, immune defects develop earlier in mucosal tissues, especially in the gastrointestinal tract and associated lymphoid organs like the gut draining mesenteric lymph nodes (MLN) [2, 3]. For example, the ability to induce oral tolerance can be lost as early as 6–8 months of age [4–6]. As most pathogens enter the body through barrier tissues, impaired mucosal immune responses, in combination with other age-related changes, are thought to account for the increased susceptibility of the elderly to infections [7]. *Listeria monocytogenes* (*Lm*) is one of the deadliest enteropathogenic bacteria, leading to the death of more than 20% of infected individuals ≥65 years old [7]. Similarly, most studies show an increased susceptibility of aged mice of different genetic backgrounds to i.v. *Lm* infection or repeated oral *Lm* gavage compared to adult mice [8–11]. Although *Lm* infection in humans occurs via the consumption of contaminated food, it remains unknown whether aging would lead to increased susceptibility of mice to naturally acquired *Lm* infection.

It is generally accepted that conventional αβ T cell responses are blunted and/or altered in aged individuals. The deterioration of αβ T cell responses has been suggested to rely on a combination of T cell intrinsic defects and changes in extrinsic factors such as impaired dendritic cell maturation, an altered environment in inductive sites, and increased progeronic factors [1, 12]. As a result, impaired conventional T cell responses can be readily observed during primary and memory responses [1, 8]. Despite this knowledge, current vaccination regimens primarily target the induction of robust conventional T and B cell memory populations and strategies correcting for the immune defects driven by aging are only slowly emerging. Therefore, there is a pressing need for new vaccine strategies that demonstrate efficacy in elderly populations.

Although most studies have focused on conventional T cell responses to infectious agents and vaccines, mucosal tissues are also patrolled by a myriad of unconventional lymphocytes that provide critical functions. γδ T cells are unconventional lymphocytes highly enriched in and adapted to epithelial and mucosal tissues, where they participate in multiple tissue processes during homeostasis and disease [13, 14]. In addition to providing rapid effector responses, γδ T cells also form adaptive memory populations in barrier tissues of young adult mice in response to infection or inflammation [15–21]. Indeed, our group demonstrated that foodborne *Lm* infection elicits the generation of an adaptive intestinal resident memory Vγ4Vδ1 T cell population (Garman Vγ TCR nomenclature [22]) characterized by a CD44^hi^ CD27^neg^ phenotype and a broad bacterial reactivity to intestinal pathogens [15, 23]. This memory subset has the unusual ability to co-produce IL-17A and IFNγ and participates with conventional T cells in anamnestic protection against reinfection in part through IL-17A production [15, 16]. However, our understanding of γδ T cell biology lags far behind conventional T cells in many aspects, including memory responses, infection, and aging. Observational studies reported an overall decrease in γδ T cell numbers [24–26], a shift from a naïve to a late differentiated phenotype, and a decreased proliferative capacity of human circulating γδ T cells in older individuals, although circulating Vδ2^+^ T cells seem more resistant to immunosenescence than other γδ T cell subsets [26, 27]. More recently, an age-dependent accumulation of IL-17A-producing γδ T (γδT_17_) cells has been shown in mouse adipose tissues [28], lungs [29] and lymphoid tissues [30]. As such, some γδ T cell subsets may be more resistant to the deleterious effects of aging and may provide exploitable anti-infectious functions in aged hosts or represent targetable cellular subsets in inflammatory diseases. Therefore, we decided to evaluate the effect of aging on the formation and function of mucosal adaptive Vγ4 T cells elicited by foodborne *Lm* infection of C57BL/6 (B6) and Balb/c mice.

## Results

### An increased CD44^hi^ CD27^neg^ Vγ1.1^neg^ Vγ2^neg^ γδ T cell response to foodborne *Lm* infection with aging

**Fig. 1:**
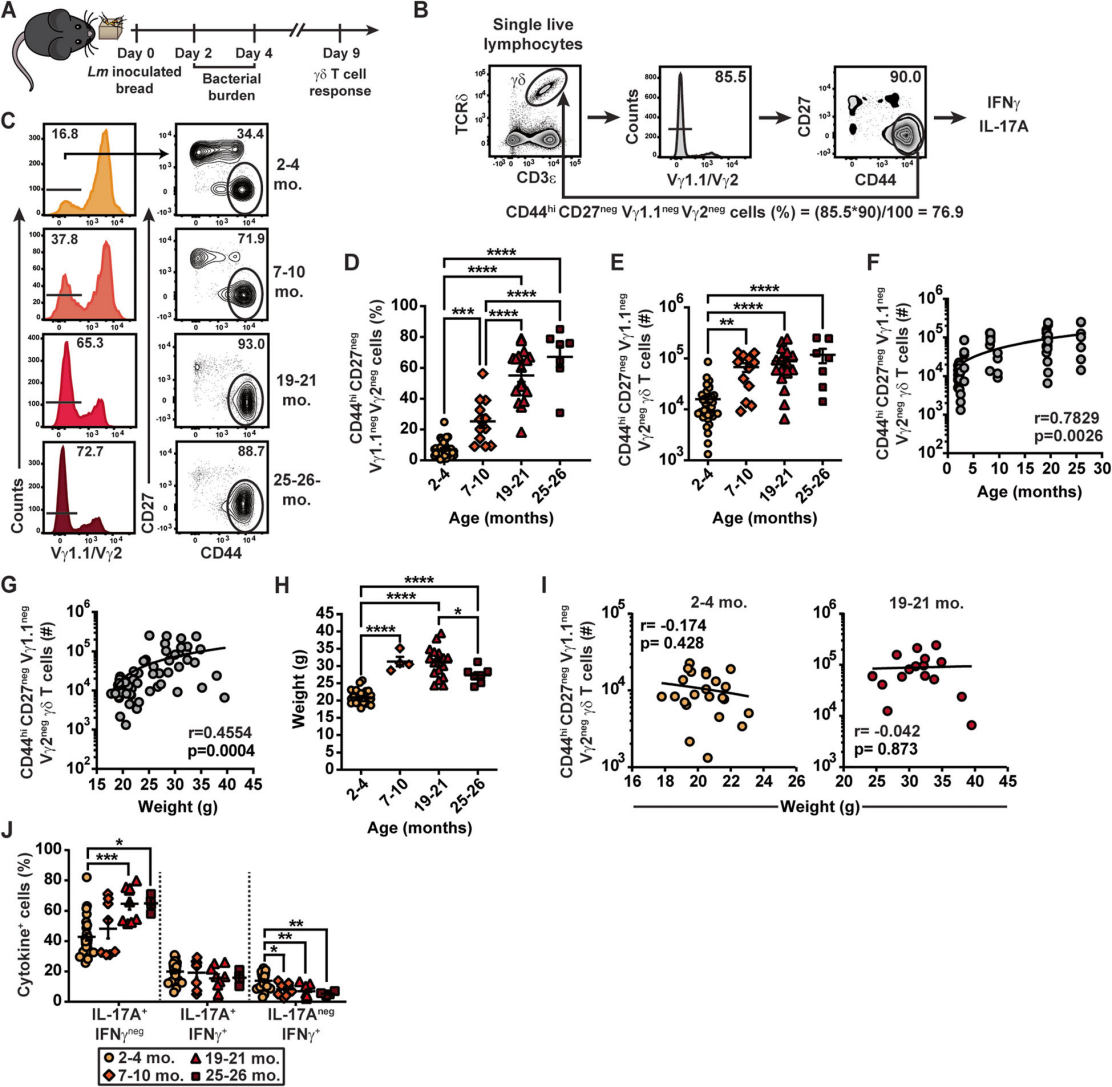
Foodborne*Lm* infection elicits a robust and diverse CD44^hi^ CD27^neg^ Vγ1.1^neg^ Vγ2^neg^ γδ T cell response with aging. B6 mice were foodborne infected with OVA-expressing *Lm.* MLN were harvested and CD44^hi^ CD27^neg^ Vγ1.1^neg^ Vγ2^neg^ γδ T cells were analyzed by flow cytometry 9 days later. **A** Schematic of the experimental protocol. **B** Gating strategy used for the identification and calculation of the frequency of CD44^hi^ CD27^neg^ Vγ1.1^neg^ Vγ2^neg^ γδ T cells among total γδ T cells is depicted. **C** Representative flow plots for the indicated groups are shown. Compounding frequency among total γδ T cells (**D**) and absolute numbers (**E**) of CD44^hi^ CD27^neg^ Vγ1.1^neg^ Vγ2^neg^ γδ T cells are shown as mean ± SEM. **F** Linear regression between CD44^hi^ CD27^neg^ Vγ1.1^neg^ Vγ2^neg^ γδ T cell numbers and age is shown. **G** Linear regression between CD44^hi^ CD27^neg^ Vγ1.1^neg^ Vγ2^neg^ γδ T cell numbers and weight is shown. **H** Body weight for each age group is shown as mean ± SEM. **I** Linear regressions between CD44^hi^ CD27^neg^ Vγ1.1^neg^ Vγ2^neg^ γδ T cell numbers and weight within indicated age groups are shown. **J** CD44^hi^ CD27^neg^ Vγ1.1^neg^ Vγ2^neg^ γδ T cell functional was analyzed at 9 days post-infection. Data show the mean ± SEM of cytokine-producing cells. Each symbol represents an individual mouse. All data sets are compiled from at least 3 independent experiments with a minimum of 3 mice/group, except 25-26-month-old B6 (1 experiment, 7 mice) and 7-10-month-old in (**H**) (1 experiment, 4 mice)

Unlike most γδ T cell subsets, γδT_17_ cells have recently been shown to accumulate with aging. As foodborne *Lm* infection of adult mice induces the formation of long-lived IL-17A-producing memory Vγ4 T cells [15, 16], *Lm*-elicited γδ T cells were assessed after foodborne infection of B6 mice with aging. Mice aged 2–4, 7–10, 19–21, or 25–26 months were foodborne infected with 2-3 × 10^9^ CFU of an internalin A mutant, mouse-adapted *Lm* via the consumption of inoculated bread as previously reported [15]. Since defective primary conventional T cell responses often lead to reduced memory responses [1, 8], we evaluated the impact of aging on the generation of *Lm*-elicited adaptive γδ T cells in the MLN 9 days post-infection (dpi), at the peak of the primary effector Vγ4 T cell response (Fig. 1A)[15, 23]. *Lm*-elicited adaptive γδ T cells were identified by the phenotype CD44^hi^ CD27^neg^ and expressed the Vγ4Vδ1 TCR, which were identified in the MLN using a dump gate for Vγ1.1 and Vγ2 (Fig. 1B and C and [15, 16]). This strategy was used as virtually all γδ T cells present in the mesenteric and peripheral LN express either Vγ2, Vγ4, or Vγ1.1 TCRs in young adult and aged mice [30]. Indeed, staining with the newly developed Vγ4-specific antibody 1C10-1F7 suggests that our gating strategy primarily identifies Vγ4^+^ T cells in the peripheral LN of aged mice (Additional Fig. S1), as recently reported in young adult mice [23]. Foodborne *Lm* infection of mice elicited a response from CD44^hi^ CD27^neg^ Vγ1.1^neg^ Vγ2^neg^ γδ T cells that gradually increased in frequency with mouse age (Fig. 1D), resulting in a significant increase in absolute numbers as early as 7–10 months of age which was maintained in 19-21- and 25-26-month-old mice (Fig. 1E). As adult Balb/c mice do not display major differences with B6 mice in bacterial burden or dissemination after foodborne *Lm* infection [31], we also assessed the Vγ4 T cell response in Balb/c mice. Balb/c mice also demonstrated a similarly enhanced CD44^hi^ CD27^neg^ Vγ1.1^neg^ Vγ2^neg^ γδ T cell response after foodborne *Lm* infection suggesting that this response is conserved among diverse genetic backgrounds (Additional Fig. S2A-C).

Foodborne *Lm* infection induces robust tissue-resident memory CD8^+^ T cell responses [32, 33]. We thus wondered whether intestinal antigen-specific CD8^+^ T cell responses would follow a similar trajectory as adaptive Vγ4 T cells. Because the *Lm* strain used to infect B6 mice expresses a truncated form of ovalbumin containing the immunogenic OVA_257 − 264_ epitope, we tracked CD8^+^ T cells specific for this peptide. OVA_257 − 264_-specific CD8^+^ T cell responses only showed a transient increase in frequency in the 7-10-months old groups, which was not maintained in older mice infected with OVA-expressing *Lm* (Additional Fig. S3). Thus, the impact of aging on CD44^hi^ CD27^neg^ Vγ1.1^neg^ Vγ2^neg^ γδ T cells after foodborne infection is not necessarily translatable to conventional T cells.

As aging is often associated with an increased body mass and γδ T cells accumulate in adipose tissues over time [28], the relationship between age, weight and CD44^hi^ CD27^neg^ Vγ1.1^neg^ Vγ2^neg^ γδ T cells was evaluated. While age showed a strong association with CD44^hi^ CD27^neg^ Vγ1.1^neg^ Vγ2^neg^ γδ T cell numbers in infected mice (Fig. 1F), weight demonstrated a more modest (although significant) correlation (Fig. 1G). Weight comparison between the different age groups showed that mice significantly gained weight between 2 and 4 and 7–10 months of age and that body mass was only maintained, or even decreased, afterwards (Fig. 1H). Furthermore, diverse weights did not correlate with the enhanced CD44^hi^ CD27^neg^ Vγ1.1^neg^ Vγ2^neg^ γδ T cell response among discreet age groups (Fig. 1I). Thus, increased body mass does not appear to be a major contributor to the enhanced CD44^hi^ CD27^neg^ Vγ1.1^neg^ Vγ2^neg^ γδ T cell response, although it may contribute to the early enhancement observed in 7–10 month-old mice. Together, these data suggest that some age-related factors other than body mass promote the development of CD44^hi^ CD27^neg^ Vγ1.1^neg^ Vγ2^neg^ γδ T cells during foodborne *Lm* infection in aged mice.

### CD44^hi^ CD27^neg^ Vγ1.1^neg^ Vγ2^neg^ γδ T cell ability to produce IFNγ and IL-17A is mostly preserved in infected aged mice

*Lm*-elicited CD44^hi^ CD27^neg^ Vγ1.1^neg^ Vγ2^neg^ γδ T cells form a functionally heterogeneous population of cells that encompasses IFNγ- and IL-17A-producing cells and cells capable of co-producing IFNγ and IL-17A [15]. Because aging may lead to an enrichment in γδT_17_ cells [28–30], the function of *Lm*-elicited CD44^hi^ CD27^neg^ Vγ1.1^neg^ Vγ2^neg^ γδ T cells was assessed. The frequency of IL-17A-producing cells increased gradually as B6 and Balb/c mice aged (Fig. 1J and Additional Fig. S2D), consistent with the accumulation of γδT_17_ cells previously reported in other tissues [28–30]. A corresponding, age-dependent reduction in IFNγ-producing cells was observed. However, IL-17A and IFNγ co-producing cell frequency was comparable in all age groups, suggesting that aging did not affect this functional subset (Fig. 1J and Additional Fig. S2D). Thus, aging elicits a modest functional shift in *Lm*-elicited CD44^hi^ CD27^neg^ Vγ1.1^neg^ Vγ2^neg^ γδ T cells toward IL-17A production resulting in a subtle but significant increase in IL-17A-producing cells. Despite this change, these data suggest that CD44^hi^ CD27^neg^ Vγ1.1^neg^ Vγ2^neg^ γδ T cells remain a population with diverse functional subsets in mucosal tissues during aging.

### The increased adaptive Vγ4 T cell response after foodborne *Lm* infection is not due to increased pathogen burden

**Fig. 2:**
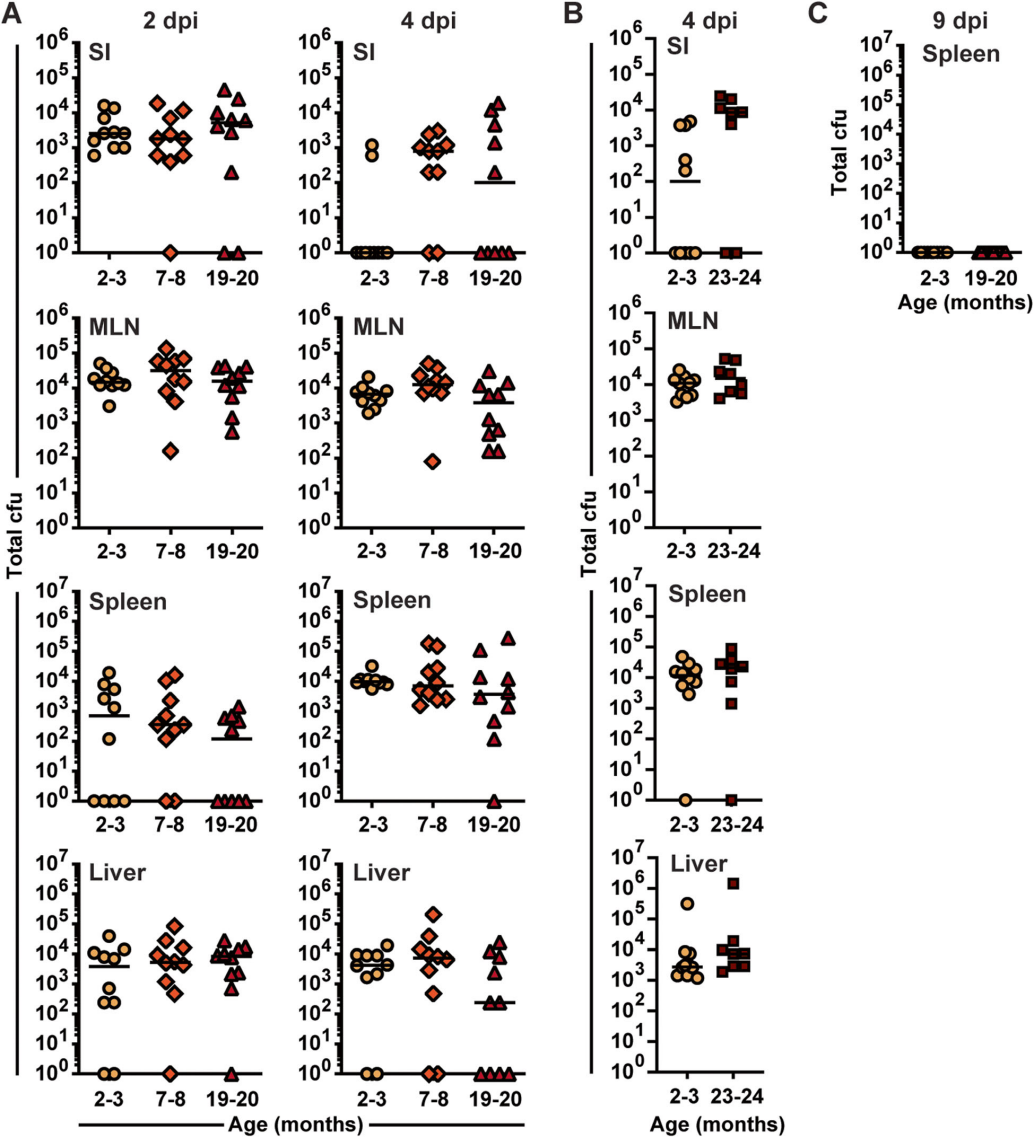
Containment of bacteria after foodborne *Lm* infection of mice with aging. B6 mice of the indicated ages were foodborne infected with the streptomycin resistant 10403s *Lm* strain. Bacterial burden was evaluated at **A** 2 and 4, **B** 4, and **C** 9 days post-infection in the indicated tissues. Combined data of 2 independent experiments with 4–6 mice per age group is shown. Both males and females were used in panel (B). Median is depicted. SI, small intestine; MLN, mesenteric lymph nodes; dpi, days post-infection

It is well established that aged mice are highly susceptible to i.v. *Lm* infection [8–10]. Furthermore, a recent study showed that aged mice are also susceptible to repeated oral gavage *Lm* infection, a model that leads to sustained systemic colonization [11]. Since the susceptibility of aged mice to a single foodborne *Lm* infection was unknown and because increased colonization may promote a more robust adaptive Vγ4 T cell response, *Lm* burden was evaluated at local and systemic sites at 2 and 4 days after foodborne infection (Fig. 1A). Based on our previously published study, *Lm* burden peaks around day 2–3 after foodborne infection, and the bacteria is largely cleared in all infected tissues between 5 and 8 days post-infection [31]. Unexpectedly, *Lm* burden was comparable between 2 and 3-, 7-10-, 19-20-, and 23-24-month-old mice in all tissues tested (Fig. 2A and B). Furthermore, *Lm* was cleared from the spleen of 2-3- and 19-20-month-old B6 mice by 9 dpi (Fig. 2C), indicating that single foodborne infection does not lead to sustained systemic colonization. Thus, B6 mice were competent to contain bacterial replication after foodborne *Lm* infection with aging. Despite this containment of bacterial replication, aged mice lost more weight and had a delayed recovery after foodborne *Lm* infection (Additional Fig. S5A and B), suggesting that some susceptibility to disease remains with aging. Collectively, these data suggest that the increased adaptive Vγ4 T cell response observed after foodborne *Lm* infection with aging is not due to increased bacterial burden.

### CD44^hi^ CD27^neg^ Vγ1.1^neg^ Vγ2^neg^ γδ T cells accumulate in intestinal and non-intestinal tissues of naïve mice with aging

**Fig. 3:**
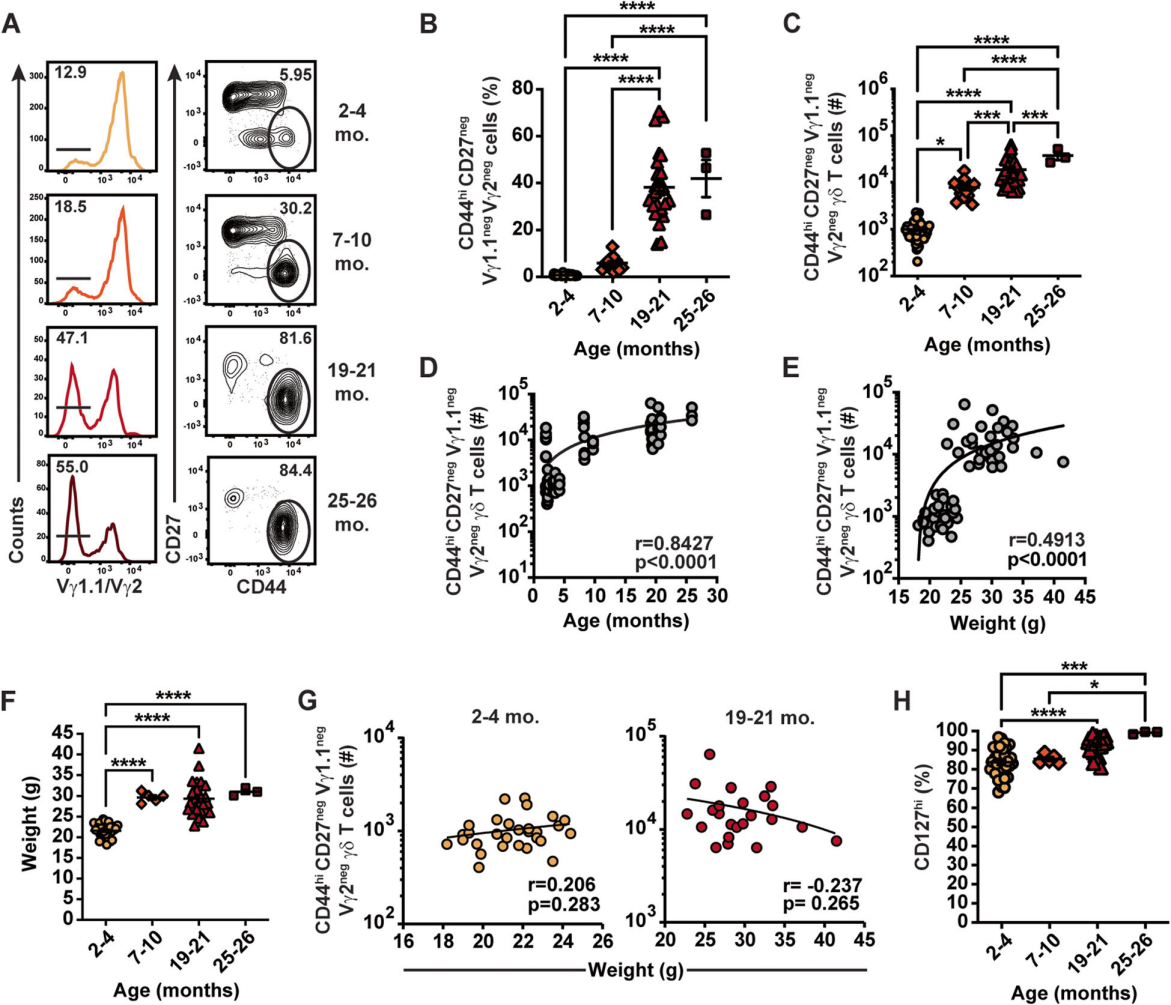
Age-associated accumulation of CD44^hi^ CD27^neg^ Vγ4 T cells in naïve mice. Vγ4 T cells in the MLN of naïve B6 mice of different ages were analyzed. **A** Representative flow plots for the indicated age groups are shown. Compounded frequency of CD44^hi^ CD27^neg^ Vγ4 T cells among total γδ T cells (**B**) and absolute numbers (**C**) of CD44^hi^ CD27^neg^ Vγ4 T cells are shown as mean ± SEM. All data sets in (**B**-**E**) are compiled from at least 3 independent experiments with a minimum of 3 mice/group, except 25-26-month-old B6 (1 experiment, 3 mice). **D** Linear regression between CD44^hi^ CD27^neg^ Vγ4 T cell numbers and age is shown. **E** Linear regression between CD44^hi^ CD27^neg^ Vγ4 T cell numbers and weight is shown. **F** Body weight for each age group is shown as mean ± SEM. **G** Linear regressions between CD44^hi^ CD27^neg^ Vγ4 T cell numbers and weight by indicated age groups are shown. **H** Frequency of CD127^hi^ cells is shown as mean ± SEM. Each symbol represents an individual mouse. All data sets in (**F**-**H**) are compiled from at least 3 independent experiments with a minimum of 3 mice/group, except 7-10-month-old B6 (1 experiment, 5 mice) and 25-26-month-old B6 (1 experiment, 3 mice)

Whether the enhancement of the *Lm*-elicited CD44^hi^ CD27^neg^ Vγ1.1^neg^ Vγ2^neg^ γδ T cell response with aging was associated with an increased precursor population was assessed by evaluating CD44^hi^ CD27^neg^ Vγ1.1^neg^ Vγ2^neg^ γδ T cells in naïve mice. A conserved, age-associated increase in CD44^hi^ CD27^neg^ Vγ1.1^neg^ Vγ2^neg^ γδ T cells was observed during normal homeostasis in the MLN of B6 and Balb/c mice (Fig. 3A-C and Additional Fig. S4A-C). Importantly, CD44^hi^ CD27^neg^ Vγ1.1^neg^ Vγ2^neg^ γδ T cell frequencies and numbers are significantly increased after foodborne *Lm* infection in all age groups analyzed up to 21 months old mice (Additional Fig. S5C and D). A trend toward an increase was also present in 25-26-month-old mice (Additional Fig. S5C and D), showing that foodborne *Lm* infection elicits a CD44^hi^ CD27^neg^ Vγ1.1^neg^ Vγ2^neg^ γδ T cell response which is increased upon aging. However, aged mice showed a reduced fold increase in total CD44^hi^ CD27^neg^ Vγ1.1^neg^ Vγ2^neg^ γδ T cell number after infection compared to young mice (Additional Table S1), suggesting that aging may lead to some proliferative defects in adaptive Vγ4 T cells that are overcome by elevated precursor Vγ4 T cell numbers. Similar to infected mice, age showed a strong positive correlation with CD44^hi^ CD27^neg^ Vγ1.1^neg^ Vγ2^neg^ γδ T cells in the MLN of naïve mice (Fig. 3D). Although body mass moderately correlated with CD44^hi^ CD27^neg^ Vγ1.1^neg^ Vγ2^neg^ γδ T cells (Fig. 3E), mice did not gain weight between 7 and 26 months of age (Fig. 3F) and low to no correlation between weights and CD44^hi^ CD27^neg^ Vγ1.1^neg^ Vγ2^neg^ γδ T cell numbers was observed among discreet age groups (Fig. 3G). As such, increased body mass does not appear to be a major contributor to the elevated precursor population. As aging does not impact the colonization or dissemination of *Lm* after foodborne infection (Fig. 2), these results collectively suggest that the accumulation of a larger precursor population associated with aging is a major driver of the robust effector CD44^hi^ CD27^neg^ Vγ1.1^neg^ Vγ2^neg^ γδ T cell responses to foodborne *Lm* infection in intestinal tissues.

γδT_17_ cell accumulation has been reported in both lymphoid and mucosal tissues of aged mice [29, 30]. This led us to assess whether the age-dependent increase in CD44^hi^ CD27^neg^ Vγ1.1^neg^ Vγ2^neg^ γδ T cells was restricted to the MLN. CD44^hi^ CD27^neg^ Vγ1.1^neg^ Vγ2^neg^ γδ T cells were significantly increased in other compartments of the digestive system including the lamina propria (LP) of the small intestine and the liver (Additional Fig. S6). Interestingly, increased precursor populations were also observed in non-intestinal barrier tissues such as the lungs, ear skin, and lymphoid tissues like the spleen and peripheral non-mesenteric lymph nodes (pLN; Additional Fig. S6). Thus, aging leads to increased CD44^hi^ CD27^neg^ Vγ1.1^neg^ Vγ2^neg^ γδ T cell populations in diverse anatomical sites. Together, these results suggest that factors with broad systemic effects drive the age-associated promotion of CD44^hi^ CD27^neg^ Vγ1.1^neg^ Vγ2^neg^ γδ T cell responses.

The microbiota influences γδ T cells as their development or activation are regulated by the microbiota in sites as diverse as the intestines [34, 35], liver [36], lungs [37] and the skin [38, 39]. *Lm*-elicited adaptive Vγ4 T cells share several features with commensal-induced γδ T cells including a CD44^hi^ CD27^neg^ CD62L^neg^ phenotype, expression of IL-1R1 and the ability to produce IL-17A, suggesting that they may be modulated by commensals [15, 16]. Therefore, we evaluated whether microbial colonization was responsible for the enhanced precursor population in aged mice. To evaluate this possibility, CD44^hi^ CD27^neg^ Vγ1.1^neg^ Vγ2^neg^ γδ T cells were compared between naïve germ-free (GF) and specific pathogen-free (SPF) B6 retired breeders that were between 11- and 14-month-old. A significant increase in CD44^hi^ CD27^neg^ Vγ1.1^neg^ Vγ2^neg^ γδ T cells was observed in retired breeder SPF mice compared to younger SPF mice from the same colony (Additional Fig. S7). This difference was not due to prior reproductive status (Additional Fig. S8A and B). GF mice had similar CD44^hi^ CD27^neg^ Vγ1.1^neg^ Vγ2^neg^ γδ T cells compared to SPF mice, suggesting that the microbiota is not necessary for the early accumulation of Vγ4 T cells in 11-14-month-old mice. Male and female mice also had similar CD44^hi^ CD27^neg^ Vγ1.1^neg^ Vγ2^neg^ γδ T cells (Additional Fig. S7 and S8C), suggesting that sex was not a major determining factor in Vγ4 T cell accumulation with age. As male mice weigh more than female mice, this observation is consistent with the notion that weight is not a major factor driving the accumulation of Vγ4 T cells with aging.

Homeostatic proliferation and survival of naïve T cells is known to rely on IL-7 [40, 41]. While impaired accessibility to key factors such as IL-7 is thought to contribute to the loss of naïve conventional T cells in the lymph nodes of aged mice [42], IL-7 has recently been suggested to drive the age-dependent accumulation of γδT_17_ in pLN [30]. A substantial fraction of CD44^hi^ CD27^neg^ Vγ1.1^neg^ Vγ2^neg^ γδ T cells expressed high levels of IL-7Rα (CD127) in the MLN of 2-4-month-old B6 and Balb/c mice (Fig. 3H and Additional Fig. S4D), suggesting that IL-7 may contribute to the homeostasis of CD44^hi^ CD27^neg^ Vγ1.1^neg^ Vγ2^neg^ γδ T cells. Interestingly, the proportion of CD127^hi^ cells gradually increased as mice aged, resulting in > 98% of CD44^hi^ CD27^neg^ Vγ1.1^neg^ Vγ2^neg^ γδ T cells expressing high levels of CD127 in 25-26-month-old B6 mice (Fig. 3H and Additional Fig. S4D). Thus, IL-7 may contribute to the accumulation of CD127^hi^ CD44^hi^ CD27^neg^ Vγ1.1^neg^ Vγ2^neg^ γδ T cells.

### anti-TCRδ and anti-IL-17A antibody treatment does not alter *Lm* dissemination and burden in aged mice

**Fig. 4:**
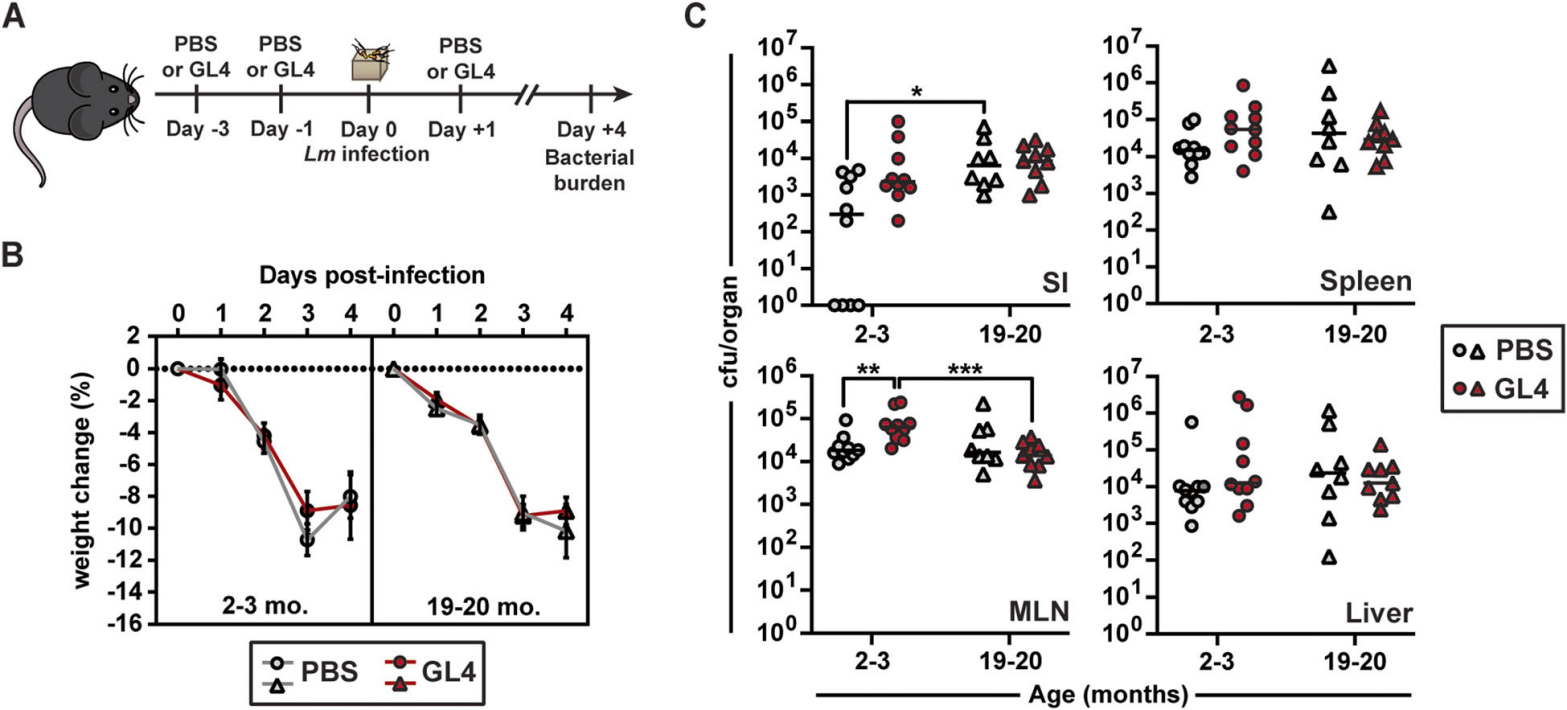
Anti-TCRδ antibody treatment of mice does not abrogate protection after foodborne *Lm* infection. 2-3- and 19-20-month-old B6 mice received 3 i.p. injections of either PBS or anti-TCRδ antibody (GL4; 100 µg/injection) on days − 3, -1, + 1 relative to infection with the streptomycin-resistant 10403s *Lm* strain. On day 0, mice were foodborne infected with *Lm*. **A** Schematic of the experimental protocol. **B** Mice were followed daily for weight loss. **C** Bacterial burden was evaluated 4 days post-infection in the indicated tissues. Data are compiled from 2 independent experiments with 4–5 mice/group

As *Lm*-inexperienced CD44^hi^ CD27^neg^ Vγ1.1^neg^ Vγ2^neg^ γδ T cells accumulated in many tissues colonized by *Lm* during foodborne infection, we evaluated whether the increased CD44^hi^ CD27^neg^ Vγ1.1^neg^ Vγ2^neg^ γδ T cell response in 19–20-month-old mice contributes to the control of *Lm* replication. To test this possibility, 2-3- and 19-20-month-old B6 mice were treated with the anti-TCRδ antibody clone GL4 or PBS as control (Fig. 4A). Treatment with anti-TCRδ antibodies induces the internalization of the TCR but does not deplete γδ T cells (Additional Fig. S9 and [15, 43]). This approach was chosen as GL4 treatment of immunized young adult mice, in combination with CD4 and CD8 T cell depletion, results in a loss of protection upon challenge *Lm* infection [15]. PBS- and GL4-treated mice had a comparable weight loss from 0 to 3 dpi and all groups stabilized weight similarly at 4 dpi (Fig. 4B), suggesting that the treatment did not affect the course of the infection. In line with this observation, *Lm* burden was similar between control and GL4-treated mice, regardless of their age, except for the MLN in young mice (Fig. 4C). These results suggest that the γδTCR may not be required for the early control of *Lm* replication in 2-3- and 19-20-month-old mice.

Protective Vγ4 T cell responses mediated by Vγ1.1^neg^ Vγ2^neg^ and Vδ1^+^ T cells have been reported within a few days of intraperitoneal (i.p.) *Lm* infection of young adult B6 mice [44, 45]. Control of bacterial replication was dependent on IL-17A production in the peritoneum and liver [44, 46]. Similarly, *Lm*-elicited memory Vγ4 T cells are the primary source of IL-17A one day after *Lm* challenge infection of previously immunized young adult mice, which promotes the control and clearance of *Lm* [16]. As CD44^hi^ CD27^neg^ Vγ1.1^neg^ Vγ2^neg^ γδ T cells remained composed of diverse functional subsets with aging, we evaluated whether neutralization of IL-17A would diminish the resistance of aged mice to foodborne *Lm* infection. However, we did not observe any significant difference between IgG1 control- and αIL-17A-treated mice regardless of age (Additional Fig. S10). As such, IL-17A does not appear to be necessary in the early control of *Lm* after primary foodborne infection in young adult and aged mice.

## Discussion

It is generally accepted that aging leads to defective immune responses and increased infection-associated morbidity and mortality. In contrast, we show in this study that, while antigen-specific CD8^+^ T cells elicited by foodborne *Lm* infection remained stable or decreased over time, aging unexpectedly led to an increased infection-elicited mucosal adaptive Vγ4 T cell response in mice. The enhancement in Vγ4 T cell numbers appeared mainly driven by the age-dependent accumulation of a precursor population of CD44^hi^ CD27^neg^ Vγ4 T cells in naive mice as some defects were observed in the fold increase of Vγ4 T cells after foodborne infection. Importantly, the population of *Lm*-elicited adaptive Vγ4 T cells was composed of diverse functional subsets with aging despite some modest alterations promoting IL-17A production, suggesting that they may provide important effector functions in aged hosts. As such, CD44^hi^ CD27^neg^ Vγ4 T cells seem less impacted by senescence and a potential therapeutic target for anti-pathogen immunity at barrier surfaces in the elderly. While this study did not uncover a role for the γδ TCR or IL-17A in protection against primary foodborne *Lm* infection, we have previously demonstrated in young adults that *Lm*-elicited memory Vγ4 T cells provide protection to subsequent infections [15, 16] and that memory Vγ4 T cell population is composed of cells that are broadly reactive to diverse enteric pathogens [23]. As such, memory Vγ4 T cells may provide a potential target for universal vaccines against enteric pathogens in the gastrointestinal tract of elderly individuals. Alternatively, the accumulation of γδT_17_ cells with aging may promote inflammatory diseases of barrier tissues. Understanding the contribution of Vγ4 T cells to inflammatory diseases in aging may also provide opportunities for novel therapeutic interventions.

Murine Vγ4 T cells bear some resemblance to human Vδ1^+^ T cells. Vδ1^+^ T cells only represent a small fraction of circulating lymphocytes while predominating in mucosal tissues. Generally, Vδ1^+^ T cells are considered more adaptive-like. For example, they display characteristics more commonly associated with memory responses seen in conventional T cells, undergo antigen-driven clonal expansion, and transition from naïve to effector phenotypes [47–50]. Functionally, Vδ1^+^ T cells are primarily associated with production of IL-17A but can also produce IFNγ [13]. Importantly for the context of this study, Vδ1^+^ T cells numbers are maintained or expanded with age [25, 51]. Murine Vγ4^+^ T cells also bear some resemblance to a more recently identified adaptive-like subset of Vγ9^−^Vδ2^+^ T cells that also undergoes clonal expansion and differentiation [52]. Thus, understanding the biology of Vγ4 T cells may provide insights into the aging of human γδ T cells.

Although differences in mucosal immune functions have been reported between mouse strains [53, 54], the enhanced CD44^hi^ CD27^neg^ Vγ4 T cell response described here was conserved between B6 and Balb/c mice. This suggests that the factors promoting the accumulation of precursor cells and expansion of effector cells in aged mice are relatively conserved. IL-7 is a robust candidate given the conserved high expression of CD127 on precursor CD44^hi^ CD27^neg^ Vγ4 T cells in naive B6 and Balb/c mice, and the association of IL-7 with γδT_17_ cell proliferation in LN [30, 55]. Changes in the architecture of secondary lymphoid organs in aged mice, including the MLN, results in the improper recruitment and localization of conventional T cells within the tissue, leading to reduced accessibility to survival factors such as IL-7 and decreased survival and proliferation [42]. In contrast, γδT_17_ cells were observed near IL-7-rich areas in the LN of aged mice [30], suggesting that they may have some migratory advantages over CD8^+^ T cells. *Lm*-elicited adaptive Vγ4 T cells are primarily located in the medullary and the interfollicular areas of the MLN in young adult mice, not the T cell zone like αβ T cells [16]. The distinct localization of CD44^hi^ CD27^neg^ Vγ4 T cells within the LN may give them preferential access to pro-survival factors. In addition to localization issues, aged naïve conventional T cells also have impaired IL-7 signaling [42]. IL-7 signaling in γδT_17_ cells is mediated by STAT3 [55], whereas CD8^+^ and CD4^+^ T cells signal through STAT5 [42, 56]. As such, IL-7-STAT3 signaling may be preserved in aged Vγ4 T cells resulting in their accumulation. As these possibilities are not mutually exclusive, further work is needed to establish the role of tissue distribution and IL-7-STAT signaling in the age-dependent enhancement of CD44^hi^ CD27^neg^ Vγ4 T cell responses described in this study.

While evaluating the potential contribution of several factors to the enhancement of *Lm*-elicited adaptive Vγ4 T cell responses, we established that 19-20-month-old mice were as capable as young adult mice in containing *Lm* after foodborne infection. These results contrast with the reported increased susceptibility of aged mice to i.v. *Lm* infection [8–10] and invasive listeriosis elicited by repeated oral gavage [57]. Despite control of bacterial burden, aged mice displayed increased weight loss and delayed recovery after foodborne *Lm* infection suggesting some level of disease susceptibility to *Lm* remains after foodborne infection. There are also key differences that may contribute to the discrepancies between this study and others. First, foodborne infection leads to a delayed *Lm* colonization of the spleen and liver of Balb/c and B6 mice, while delivery of *Lm* through both i.v. and oral gavage elicits rapid (within 24 h) systemic bacterial dissemination to these organs [8, 10, 31, 58]. These data suggest that oral gavage may lead to unintended and rapid delivery of *Lm* to blood circulation, which is not observed with foodborne infection [31]. Second, this study was performed after a single *Lm* exposure with an infection dose that closely corresponds to the inoculum that leads to gastrointestinal disease in healthy humans on a per weight basis [59]. At this dose, most young adult mice clear *Lm* around day 7 [31]. This contrasts with the long-lasting, systemic bacterial colonization reported after repeated oral gavage in young adult and aged mice, which may contribute to their heightened susceptibility [57]. These major differences between models suggest that aged mice may be more susceptible to sustained systemic *Lm* infection than naturally acquired foodborne *Lm* infection.

Protective memory Vγ4 T cells are elicited by foodborne, but not i.v., infection of young adult mice [15]. As such, the induction of robust adaptive Vγ4 T cell responses could contribute to the difference in susceptibility of aged mice to different infection models. Therefore, we evaluated whether the unexpected resistance of 19-20-month-old or older mice to foodborne *Lm* infection stems from the increased adaptive Vγ4 T cell response. *Lm*-elicited memory Vγ4 T cell-mediated protection against challenge *Lm* infection in immune young adult mice can be mediated by both IL-17A production [16] and a γδTCR-dependent mechanism [15]. However, neither neutralization of IL-17A nor internalization of the γδTCR affected the early control of *Lm* dissemination and replication in 2-3- and 19-20-month-old or older mice during primary infection. Although these results support the possibility that CD44^hi^ CD27^neg^ Vγ4 T cells do not participate in protection after primary foodborne infection, several factors may also contribute to these observations. While the absence of a role for the γδTCR during primary infection may not be unexpected given the potentially detrimental impact of T cells on early immune control of *Lm* and the presence of an intact innate immune compartment [15, 60], the efficient control of *Lm* burden upon IL-17A neutralization is surprising. Indeed, aged IL-17RA-deficient mice were shown to be more susceptible to infection upon oral gavage with *Lm* compared to WT mice [61]. However, several members of the IL-17 family signal through IL-17RA [62]. Thus, cytokines such as IL-17F may contribute to the resistance of aged mice to *Lm*. On the other hand, IL-17A may not be required during primary foodborne infection. Indeed, protective roles for Vγ4 T cells were shown upon intraperitoneal primary infection [44, 46] or challenge infection of *Lm*-immune mice with high inoculum [15], which results in a higher proportion of extracellular bacteria [31, 63]. Foodborne infection with 2 × 10^9^ CFU of InlA^M^ *Lm* does not lead to extracellular replication except to some degree in the liver [31]. Thus, it is also possible that CD44^hi^ CD27^neg^ Vγ4 T cells use other mechanisms of protection dependent on the dynamic nature of the *Lm* lifecycle and that IL-17A promotes control of extracellular *Lm* only. Additional work is needed to evaluate these possibilities and determine whether CD44^hi^ CD27^neg^ Vγ4 T cells participate in bacterial control after primary foodborne *Lm* infection.

Recently, the accumulation of IL-17A-producing γδ T cells has been associated with beneficial or detrimental effects depending on the tissue [28, 30]. As γδ T cells may represent a major source of IL-17A, it remains to be established whether they promote immunopathology in the intestines and associated tissues with aging. A better understanding of the processes controlling mucosal T cell responses with aging is critical for the development of safe, effective vaccine-induced immunity in the elderly.

## Conclusions

Although the detrimental effects of aging on conventional T cells is well established, its impact on unconventional T cells such as γδ T cells has been less studied. We built on our previous observations that intestinal Vγ4 T cells can form memory populations upon foodborne *Lm* infection in young adult mice to evaluate whether and how aging affects their response. The present study demonstrates that adaptive Vγ4 T cells are increased and largely functional in mice of different genetic backgrounds with aging. Selective accumulation of naive Vγ4 T cells appeared to be the main mechanism leading to the increased *Lm*-elicited Vγ4 T cell responses described here. As such, adaptive Vγ4 T cells appear resistant to immunosenescence and may provide a therapeutic target for vaccination strategies tailored to the elderly.

## Methods

### Mice

Only female mice were used in this study, except where otherwise stated. All Balb/c mice were obtained from the Jackson Laboratory. 7–10 months Balb/c mice were either retired breeders or received at 8–10 weeks of age and aged at Stony Brook University. 19–22 months Balb/c mice were received at 8 weeks old and aged internally. For B6 mice, 7–10 months animals were either retired breeders obtained from the Jackson Laboratory or NCI or aged internally. 2–4 months old control mice were obtained from the same source as the aged group. B6 mice aged 19–21, 23–24 and 25–26 months were from the NIA and compared to 2–4 months old B6 mice from the Jackson Laboratory. Prior reproductive status or vendor did not impact measured immune responses (Additional Fig. S6). MLN from germ-free B6 retired breeders were kindly provided by Dr. Gregory Sonnenberg (Weill Cornell Medicine). SPF B6 retired breeders (*Tcrd*-H2B-eGFP, kindly provided by Drs. Bernard Malissen and Immo Prinz) were bred and maintained at Stony Brook University. Mice were euthanized by CO_2_ inhalation. All animal experiments were performed following the Stony Brook University Institutional Animal Care and Use Committee and National Institutes of Health guidelines.

### Bacteria and infection

For bacteria burden experiments, *Lm* strain 10403s (naturally resistant to streptomycin) was used. When T cell responses were analyzed, *Lm* strain EGDe was used to infect Balb/c mice whereas B6 mice were infected with *Lm* strain 10403s expressing a truncated form of ovalbumin to allow for the evaluation of OVA_257 − 264_-specific CD8^+^ T cell responses. All *Lm* strains used express a recombinant internalin A protein carrying S192N and Y369S mutations. All mouse infections were performed orally by feeding mice bread inoculated with 2–3 × 10^9^ CFU *Lm* as previously described [15]. All infection doses were confirmed by enumerating CFU of the inoculum.

### Antibody treatment

Mice were either injected intraperitoneally with 100 µg of anti-TCRδ antibody (clone GL4) or 100 µl of PBS on days − 3, -1 and + 1, or 200 µg of anti-IL-17A (17F3) or mouse IgG1 (MOPC-21) relative to foodborne *Lm* infection. All antibodies were obtained from Bio X Cell.

### Organ burden

MLN, spleens and livers were mechanically dissociated through a 70 μm filter. Small intestines were mechanically dissociated using a gentleMACS Dissociator (Miltenyi). All samples were treated with 1% saponin (EMD Millipore) for at least 1 h at 4 °C before plating. Serial dilutions were plated on Brain Heart Infusion agar plates supplemented with 50 µg/ml streptomycin. Individual colonies were counted after 24–48 h at 37 °C.

### Leukocyte isolation

MLN, spleen and peripheral lymph nodes were harvested and mechanically dissociated into single-cell suspensions using 70 μm cell strainers. Liver was mashed through 70 μm cell strainers and leukocytes isolated using a 44–67% percoll gradient. For skin cells, the ventral and dorsal sides of the hairless part of the ears were separated from cartilage and processed as described below. For each mouse, skin tissues from both ears were pooled. Skin and lungs were cut into small pieces and digested with 100 U/ml of collagenase (Invitrogen) for 45 min at 37 °C under 220 rpm agitation. Remaining pieces were mashed through 70 μm cell strainers and combined with digested supernatants. Leukocytes were isolated using a 44–67% percoll gradient. Lamina propria leukocytes were isolated as previously described [64, 65]. Viable cells were counted with the use of a Vi-CELL Viability Analyzer (Beckman Coulter).

### Flow cytometry analysis

Cells were stained with the antibodies listed in Supporting Information Additional Table S2, live/dead dye (Thermo Fisher Scientific) and anti-CD16/CD32 (Bio X Cell) for 20 min at 4 °C in the dark. All samples were fixed with 2% paraformaldehyde for 20 min. For detection of Vγ4^+^ cells, 20 µg of 1C10-1F7 antibody was used to stain the cells prior to secondary staining with a polyclonal rat anti-mouse IgG (Invitrogen). Cells were then stained with the other conjugated antibodies. For functional analysis, MLN cells were cultured at 37 °C, 5% CO_2_ for 4 h with BD Leukocyte Activation Cocktail (BD Pharmingen) in IMDM media containing 10% FBS, 10mM HEPES, 1mM sodium pyruvate, 2mM GlutaMAX™ supplement and 1X MEM non-essential amino acids solution (Thermo Fisher Scientific). Intracellular staining was performed using BD Cytofix/Cytoperm Fixation/Permeabilization kit (BD Biosciences) according to the manufacturer’s instructions. Stained cells were acquired on a LSRFortessa (BD Biosciences). Data were analyzed with FlowJo software (TreeStar).

### Statistical analysis

Statistical analyses were performed in GraphPad Prism 9 software. Significant differences in burden in Fig. 2 were determined using Kruskal-Wallis test (3 groups) or Mann-Whitney test (2 groups). Differences in burden presented in Fig. 4 were determined using Mann-Whitney test between selected groups. Ordinary one-way ANOVA with Tukey multiple comparisons test was used to analyze T cell responses. Correlations were determined using Pearson (r) correlation. *, *p* ≤ 0.05; **, *p* ≤ 0.01; ***, *p* ≤ 0.001; ****, *p* ≤ 0.0001.

## Supplementary Information

**Additional file 1.**

## Data Availability

The datasets used and/or analyzed during the current study are available from the corresponding author on request.

## Abbreviations

*Lm*: Listeria monocytogenes
MLN: Mesenteric lymph nodes
B6: C57BL/6
pLN: peripheral lymph nodes
LPL: lamina propria leukocytes
i.p.: intraperitoneal
i.v.: intravenous
