# Supplementary material for: The accumulation of Vγ4 T cells with aging is associated with an increased adaptive Vγ4 T cell response after foodborne Listeria monocytogenes infection of mice
Source: Immun Ageing. 2022 May 3;19:19. doi: 10.1186/s12979-022-00275-y (PMC9063344; doi:10.1186/s12979-022-00275-y)
Supplement: Supplementary file 1 — Additional file 1. [file 12979_2022_275_MOESM1_ESM.docx]

**ADDITIONAL INFORMATION**

**
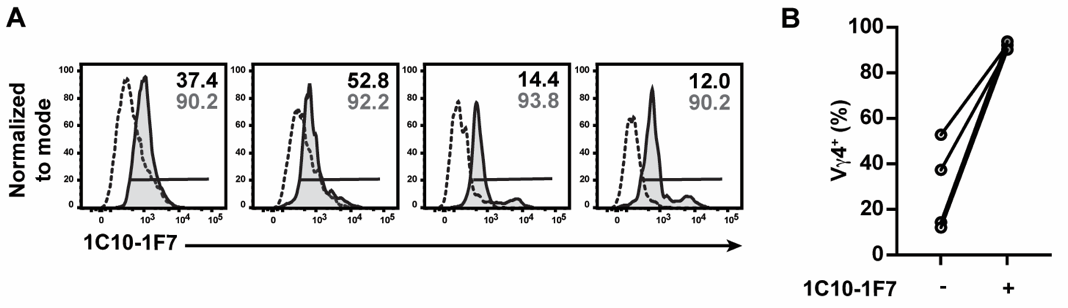
**

**Additional Figure S1. Most CD44^hi^ CD27^neg^ Vγ1.1^neg^ Vγ2^neg^ γδ T cells stain with the Vγ4-specific mAb 1C10-1F7.** 23-24-month-old C57BL/6 mice were foodborne infected with *Lm* strain 10403s. 4 days later, the inguinal lymph nodes were harvested and CD44^hi^ CD27^neg^ Vγ1.1^neg^ Vγ2^neg^ γδ T cells analyzed by flow cytometry. (A) Representative histograms of 1C10-1F7 staining (filled histograms) and control staining without primary antibody (dashed lines). Numbers indicate the percentage of cells within the depicted gates (black, no primary control; gray, 1C10-1F7 staining). (B) The frequency of 1C10-1F7^+^ cells among CD44^hi^ CD27^neg^ Vγ1.1^neg^ Vγ2^neg^ γδ T cells is shown for individual mice with or without the primary 1C10-1F7 antibody.

**
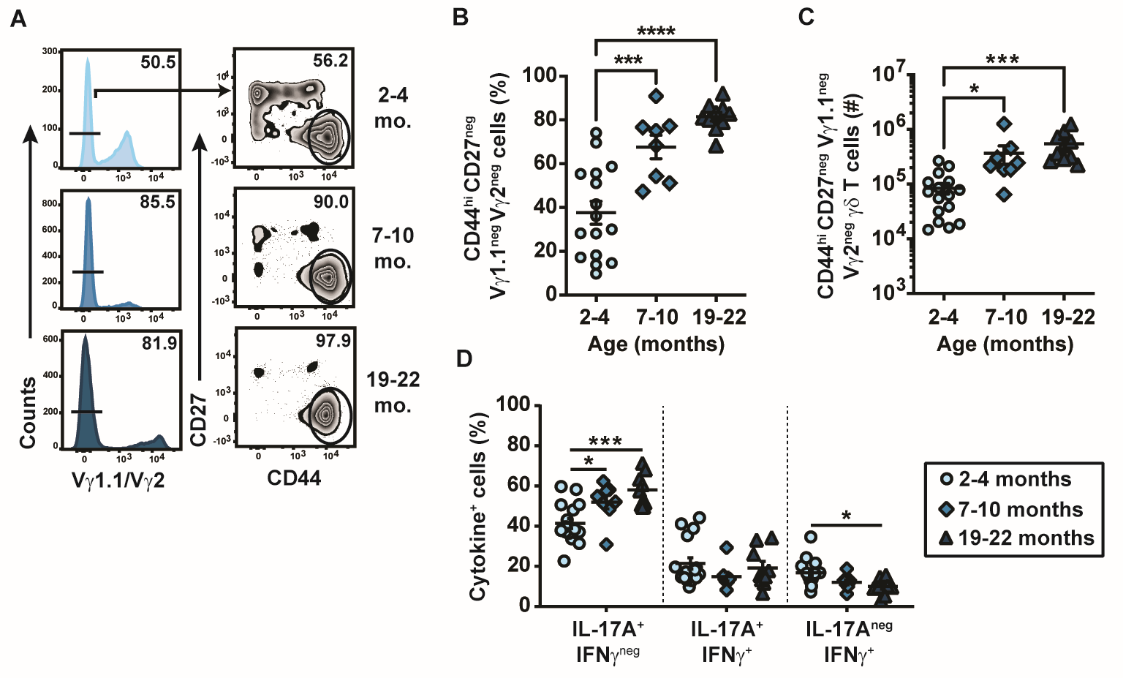
**

**Additional Figure S2. Foodborne *Lm* infection elicits a robust and functionally diverse CD44^hi^ CD27^neg^ Vγ1.1^neg^ Vγ2^neg^ γδ** **T cell response in Balb/c mice.** Balb/c mice were foodborne infected with *Lm.* MLN were harvested 9 days later and CD44^hi^ CD27^neg^ Vγ1.1^neg^ Vγ2^neg^ γδ T cells were analyzed by flow cytometry. (A) Representative flow plots for the indicated age groups are shown. (B and C) Compounded frequency of CD44^hi^ CD27^neg^ Vγ1.1^neg^ Vγ2^neg^ γδ T cells among total γδ T cells (B) and absolute numbers (C) of CD44^hi^ CD27^neg^ Vγ1.1^neg^ Vγ2^neg^ γδ T cell are shown as mean ± SEM. (D) CD44^hi^ CD27^neg^ Vγ1.1^neg^ Vγ2^neg^ γδ T cell function was analyzed at 9 days post-infection. Data show the mean ± SEM of cytokine-producing cells. All data sets are compiled from at least 2 independent experiments with a minimum of 3 mice/group. Each symbol represents an individual mouse.


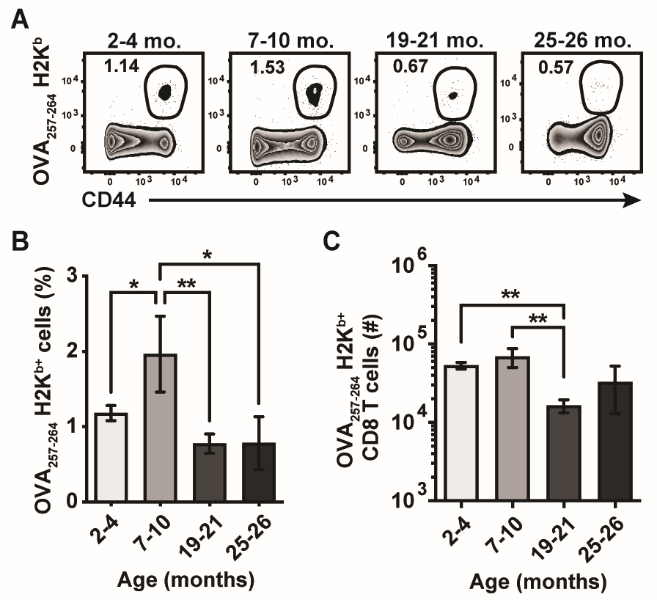


**Additional Figure S3. OVA_257-264_-specific CD8^+^ T cell responses elicited by foodborne *Lm* infection do not increase with aging.** B6 mice were foodborne infected with OVA-expressing *Lm.* MLN were harvested 9 days later and OVA_257-264_-specific CD8^+^ T cells were analyzed by flow cytometry. (A) Representative flow plots of OVA_257-274_ tetramer staining and CD44 expression among total CD8^+^ T cells for the indicated groups are shown. Frequency (B) and absolute numbers (C) of OVA_257-264_-specific CD8^+^ T cells are shown as mean ± SEM. All data sets are compiled from at least 2 independent experiments with 3-6 mice/group, except for 25-26-month-old B6 (1 experiment, 7 mice).


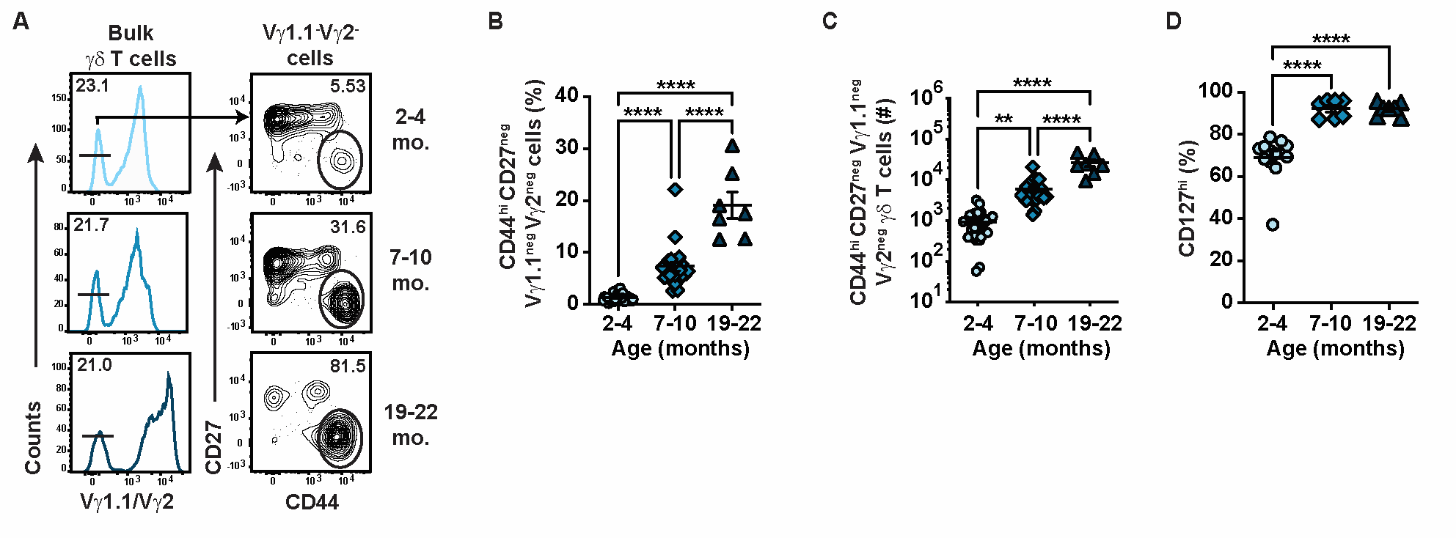


**Additional Figure S4. Age-associated accumulation of CD44^hi^ CD27^neg^ Vγ4 T cells in naïve Balb/c mice.** CD44^hi^ CD27^neg^ Vγ4 T cells in the MLN of naïve Balb/c mice of different ages were analyzed. (A) Representative flow plots for the indicated age groups are shown. Compounded frequency of CD44^hi^ CD27^neg^ Vγ4 T cells among total γδ T cells (B) and absolute numbers (C) of CD44^hi^ CD27^neg^ Vγ4 T cells are shown as mean ± SEM. (D) Frequency of CD127^hi^ cells among CD44^hi^ CD27^neg^ Vγ4 T cells are shown as mean ± SEM. All data sets are compiled from at least 2 independent experiments with a minimum of 3 mice/group. Each symbol represents an individual mouse.


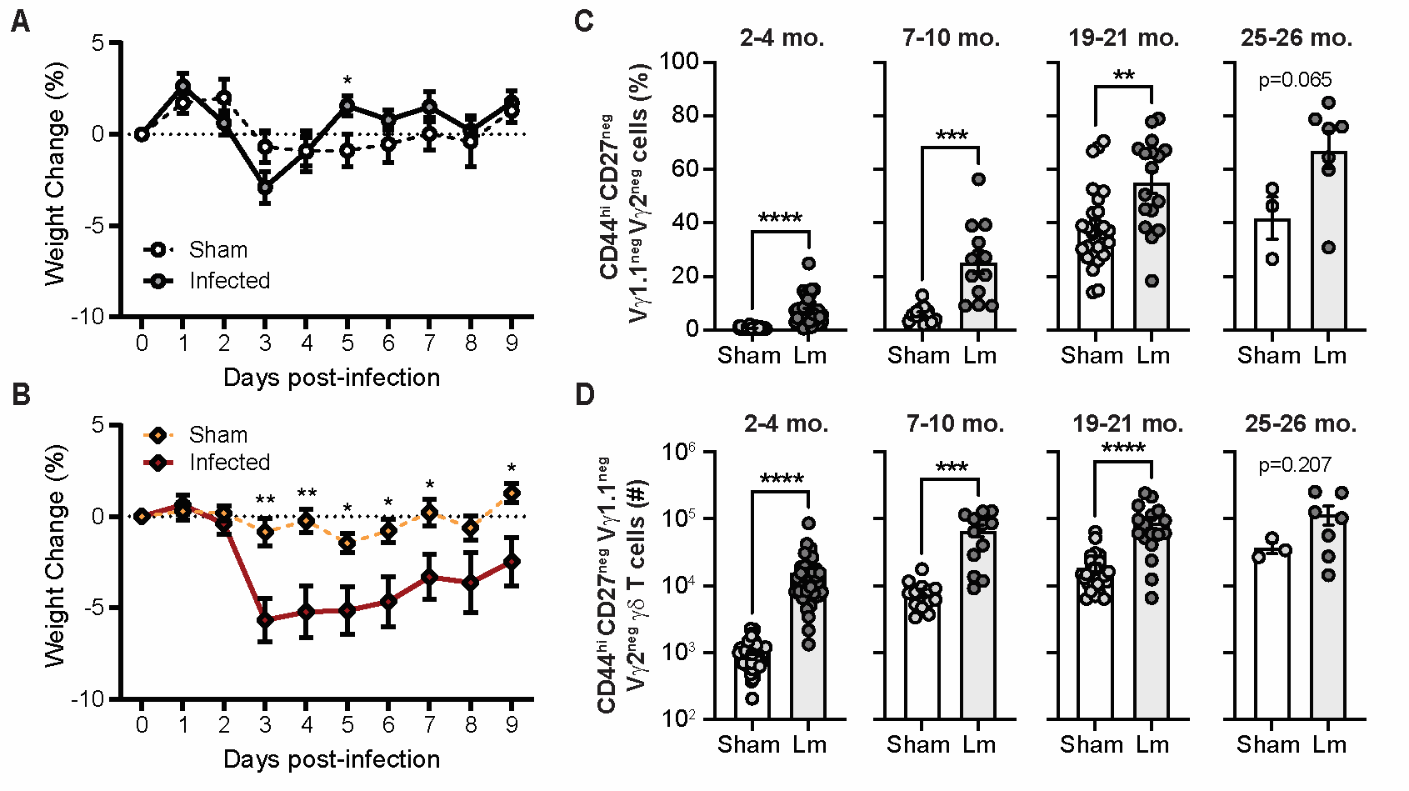


**Additional Figure S5. *Lm* elicits robust CD44^hi^ CD27^neg^ Vγ4 T cell responses in aged mice.** B6 mice of the indicated ages were foodborne infected with OVA-expressing *Lm* or were sham infected with PBS*.* (A) 2-3 month old and (B) 19 month old B6 mice were sham infected with PBS or foodborne infected with 2-3x10^9^ CFU *Lm*. Weight was recorded daily, and data are presented as percent change relative to initial weight. Graph shows the mean ± SEM pooled from 2 independent experiments (n=10 mice total, 5 mice/group/experiment). (C and D) MLN were harvested 9 days later and CD44^hi^ CD27^neg^ Vγ1.1^neg^ Vγ2^neg^ γδ T cells were analyzed by flow cytometry. Compounded frequency among total γδ T cells (C) and absolute numbers (D) of CD44^hi^ CD27^neg^ Vγ1.1^neg^ Vγ2^neg^ γδ T cells are shown as mean ± SEM. All data sets are compiled from at least 3 independent experiments with a minimum of 3 mice/group, except 25-26-month-old B6 (1 experiment, 3 to 7 mice).


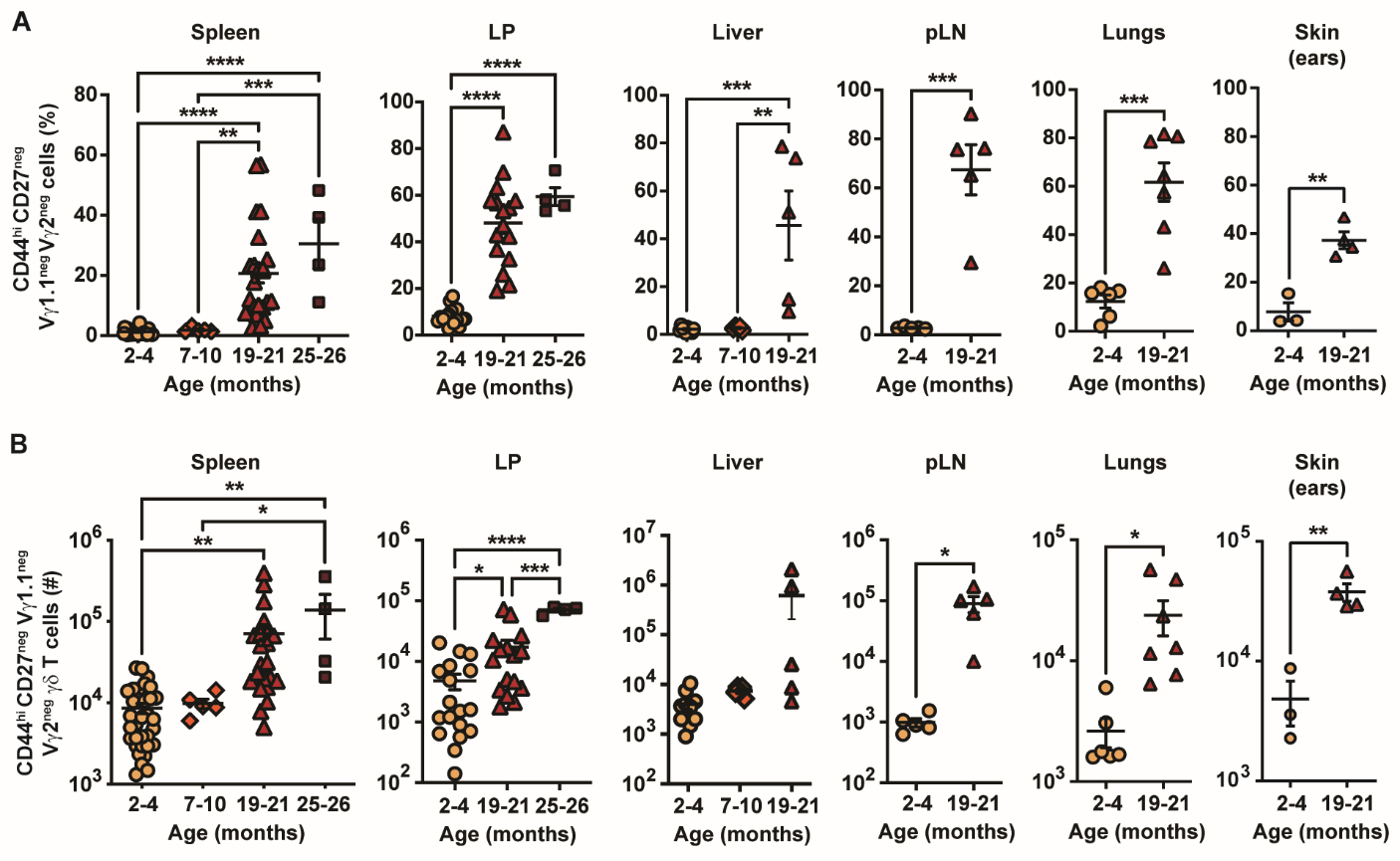


**Additional Figure S6. CD44^hi^ CD27^neg^ Vγ4 T cells accumulate in various tissues with aging during normal homeostasis.** CD44^hi^ CD27^neg^ Vγ4 T cells in the indicated tissues of naïve B6 mice of different ages were analyzed. Compounded frequency of CD44^hi^ CD27^neg^ Vγ4 T cells among total γδ T cells (A) and absolute numbers (B) of CD44^hi^ CD27^neg^ Vγ4 T cells are shown as mean ± SEM. Each symbol represents an individual mouse. LP, lamina propria; pLN, peripheral non-mesenteric lymph nodes.

**
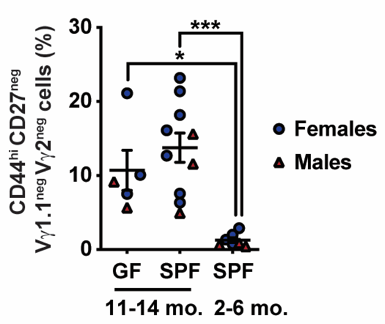
**

**Additional Figure S7. The microbiota appears dispensable for accumulation of CD44^hi^ CD27^neg^ Vγ4 T cells in naïve mice.** MLN cells from naïve 11-14-month-old retired B6 breeders maintained under germ-free (GF) or specific pathogen-free (SPF) conditions were harvested and CD44^hi^ CD27^neg^ Vγ4 T cells analyzed. 2-6-month-old SPF non-breeder B6 mice were used as a comparison. Data show the mean ± SEM of CD44^hi^ CD27^neg^ Vγ4 T cell frequency among total γδ T cells.


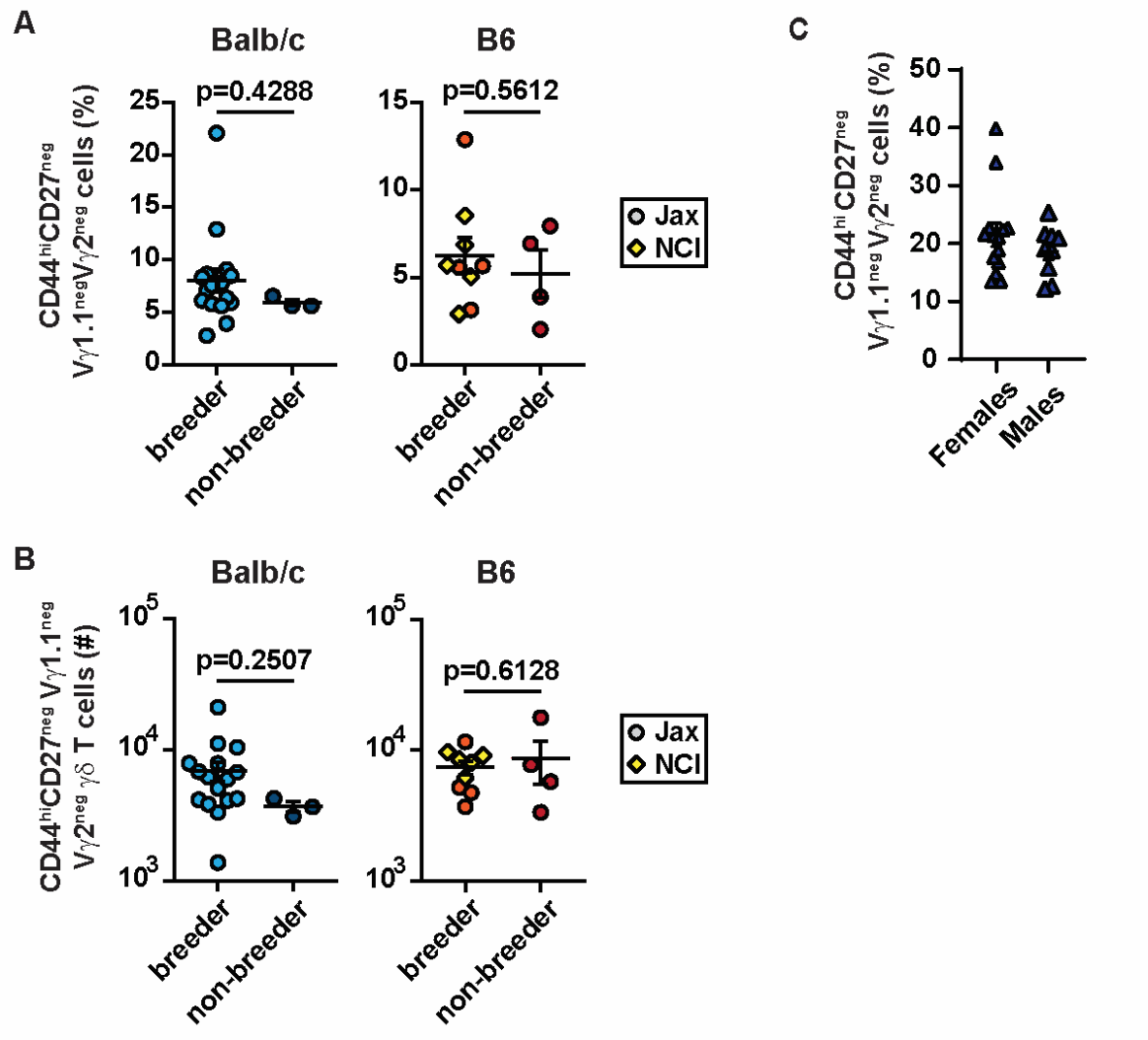


**Additional Figure S8.** **CD44^hi^ CD27^neg^ Vγ4 T cells in retired breeders, non-breeders and animals from different vendors.** MLN of naïve 7-10 month old mice were harvested and CD44^hi^ CD27^neg^ Vγ4 T cells were analyzed by flow cytometry. Compounded frequency of CD44^hi^ CD27^neg^ Vγ4 T cells among total γδ T cells (A) and absolute numbers (B) of CD44^hi^ CD27^neg^ Vγ4 T cells are shown as mean ± SEM in the scatter plots. p-values were determined using unpaired t-tests. (C) Compounded frequency of CD44^hi^ CD27^neg^ Vγ4 T cells among total γδ T cells are shown for male and female Balb/c mice that are ≥12 months old. Each symbol represents an individual mouse.


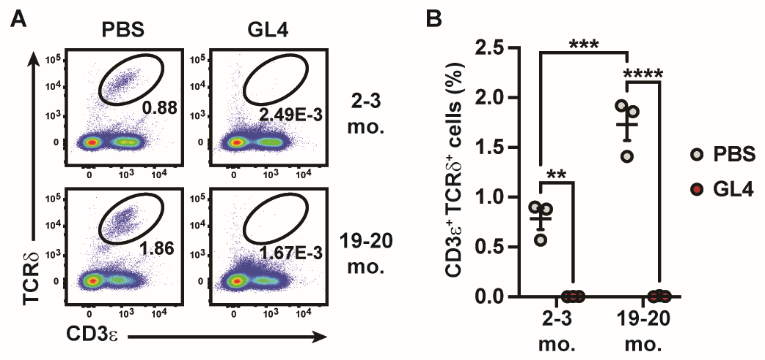


**Additional Figure S9. Surface TCR is undetectable in GL4-treated mice.** 2-3- and 19-20-month-old B6 mice were received 3 i.p. injections of either PBS or GL4 antibody (100 μg/injection) on days -3, -1, +1 relative to infection. On day 0, mice were foodborne infected with *Lm*. Presence of TCRδ^+^ cells was evaluated in the MLN 4 days later. (A) Representative flow plots of CD3ε and TCRδ expression among total live cells in the indicated groups. (B) Frequency of CD3ε^+^ TCRδ^+^ cells are shown as mean ± SEM. Data are from an experiment with 3 mice/group. Each symbol represents an individual mouse.


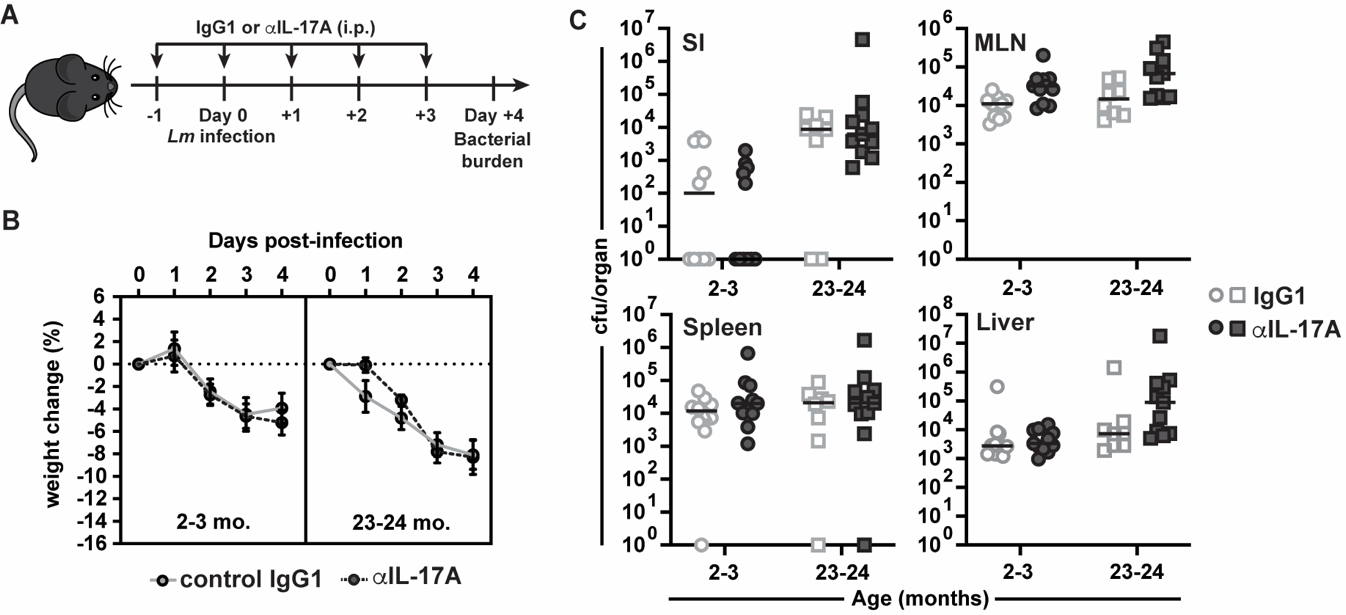


**Additional Figure S10. Protection of young adult and 23-24-month-old mice against primary foodborne *Lm* infection is unaffected by anti-IL-17A treatment.** 2-3- and 23-24-month-old B6 mice received i.p. injections of 200 μg of either control mouse IgG1 (clone MOPC-21) or anti-IL-17A antibody (clone 17F3) every day from day -1 to day +4 relative to infection with the streptomycin-resistant 10403s *Lm* strain. On day 0, mice were foodborne infected with *Lm*. (A) Schematic of the experimental protocol. (B) Mice were followed daily for weight loss. (C) Bacterial burden was evaluated 4 days post-infection in the indicated tissues. Data are compiled from 2 independent experiments with 3-6 mice/group/experiment.

|  | **# CD44^hi^ CD27^neg^ Vγ1.1^neg^ Vγ2^neg^ γδ T cells (Mean)** | **Fold increase**  ***Lm*/Naïve** |  |  |
| --- | --- | --- | --- | --- |
| **B6** | 2-4 months | Naïve | 974 | 15.3 |
|  |  | *Lm* | 14909 |  |
|  | 7-10 months | Naïve | 7794 | 8.7 |
|  |  | *Lm* | 67688 |  |
|  | 19-21 months | Naïve | 18642 | 4.8 |
|  |  | *Lm* | 88941 |  |
|  | 25-26 months | Naïve | 37467 | 3.2 |
|  |  | *Lm* | 118314 |  |
| **Balb/c** | 2-4 months | Naïve | 924 | 54.0 |
|  |  | *Lm* | 49916 |  |
|  | 7-10 months | Naïve | 5911 | 61.7 |
|  |  | *Lm* | 364700 |  |
|  | 19-21 months | Naïve | 28257 | 16.6 |
|  |  | *Lm* | 470122 |  |

**Additional Table S1. Fold change in CD44^hi^ CD27^neg^ Vγ1.1^neg^ Vγ2^neg^ γδ T cell numbers upon *Lm* infection.**

**Additional Table S2 – List of antibodies used in this study**

| Marker | Conjugate | Clone | Source |
| --- | --- | --- | --- |
| anti-mouse IgG | FITC | Poly4060 | BioLegend |
| Mouse IgG1 isotype | Purified | MOPC-21 | Bio X Cell |
| CD3ε | PE/Cy7 | 145-2C11 | BioLegend |
| CD3ε | BV421 | 145-2C11 | BioLegend |
| CD8𝛼 | PE | 53-6.7 | BioLegend |
| CD8𝛼 | BV785 | 53-6.7 | BioLegend |
| CD16/CD32 | Purified | 2.4G2 | Bio X Cell |
| CD27 | PerCP/Cy5.5 | LG.3A10 | BioLegend |
| CD44 | APC-eFluor 780 | IM7 | eBioscience |
| CD127 | PE/dazzle^TM^ 594 | A7R34 | BioLegend |
| IFN𝛾 | PE/Cy7 | XMG1.2 | BioLegend |
| IL-17A | APC | TC11-18H10.1 | BioLegend |
| IL-17A | Purified | 17F3 | Bio X Cell |
| TCR𝛽 | BV711 | H57-597 | BioLegend |
| TCRδ | BV421 | GL3 | BioLegend |
| TCRδ | PE | GL3 | BioLegend |
| TCRδ | Purified | GL4 | Bio X Cell (custom) |
| Vγ1.1 | FITC | 2.11 | BioLegend |
| Vγ1.1 | PE | 2.11 | BioLegend |
| Vγ2 | FITC | UC3-10A6 | BioLegend |
| Vγ2 | PE | UC3-10A6 | BioLegend |
| Vγ4 | Purified | 1C10-1F7 | Provided by Drs. Hatano and Yoshikai |
